# Role of primary care providers in a pandemic – conflicting views and future opportunities

**DOI:** 10.1186/s13584-015-0054-3

**Published:** 2015-11-20

**Authors:** Sarah J. Clark

**Affiliations:** Child Health Evaluation and Research (CHEAR) Unit, University of Michigan, 300 N Ingalls, Rm 6E06, Ann Arbor, MI 48109-5456 USA

**Keywords:** Primary care, Pandemic influenza, Public health

## Abstract

In pandemic situations, primary care providers may be involved in a variety of roles related to disease surveillance, diagnosis and treatment, prevention, and patient education. This commentary describes the contextual factors that may influence primary care providers’ perspectives on their pandemic roles and responsibilities. These factors include the natural evolution of the pandemic situation, with early uncertainty affecting decision-making and communication; the variation in typical practice patterns and clinical expertise across and within primary care providers; and the lack of representation of practicing primary care providers in pandemic planning and decision-making bodies.

## Background

In an innovative paper from the viewpoint of providers, Kunin and colleagues [1] offer a unique international perspective on the challenges—and contradictions--of addressing public health and emergency preparedness goals in the primary care setting. The findings of these interviews with primary care providers in three countries illustrate several common factors that affected primary care providers in these three countries, and likely in many others; findings also point to unique challenges related to specific policy choices made by these three countries regarding the role of primary care in the pandemic response.

## Main text

A common factor underlying the perspectives of primary care providers in these three countries is the inherent mismatch between the uncertainty of a pandemic situation and the desire for clear and consistent roles and responsibilities for providers. The dynamics of public health response change over the arc of a pandemic [2], though this is not always recognized at the provider level. In the earliest stage, public health policy-making is *in process*, based on the best available evidence about disease severity, availability and effectiveness of therapeutic and preventive measures, and capacity for care delivery. Policies frequently change, as new information emerges. In later stages of a pandemic, when the severity and disease patterns of the pandemic are better known, public health policies become more stable. However, many primary care providers have limited experience with this natural arc of a pandemic, and expressed frustration with what seemed to be constant policy changes and mismatches between public health policies and disease patterns and/or patient care preferences.

Another common factor is that the dynamics of communication change over the arc of a pandemic [2]. The earliest stage is a time of intense interest among the public and the media, as well as a critical juncture for informing health care professionals about public health policies and recommendation. From the public health perspective communication must be frequent and disseminated through numerous channels, to ensure that all stakeholders have an opportunity to receive the most up-to-date information. In later stages, as policies become more stable and the media attention wanes, communication can be less frequent. However, many primary care providers in this study expressed frustration with too-frequent and seemingly redundant communication of the early stage. Unlike hospital-based providers who tend to have a well-articulated chain-of-command protocol for emergency situations, primary care providers in many countries are more diffuse organizationally, geographically, and with regard to emergency contact methods; as a result, public health officials may not be able to identify a single pathway for efficient communication with all primary care providers. Moreover, many primary care practices have not designated a formal plan or process for monitoring public health communications and updating staff as needed. If responsibility for monitoring communication is not designated to certain staff, there is likely to be duplicated effort; if there is uncertainty over which communication channels will provide accurate and up-to-date information, there is likely to be unnecessary time spent sorting through communications from a broad set of organizations. As a result, it is not unexpected that primary care providers would feel overwhelmed with the pace and amount of communication at the early stage of a pandemic.

Importantly, while the article presents many provider criticisms of pandemic policies, it is clear that there was a variable response of primary care providers to pandemic policies, and no singular view of an appropriate primary care role in a pandemic situation. This variation is likely due to differences in practice patterns and clinical experience of primary care providers across and within the three countries. For example, primary care providers offered conflicting views on policies requiring them to use a centralized clinical or public health authority for tasks typically performed in the primary care setting. While some providers disparaged their country’s requirement to get approval for antiviral medications, arguing that primary care providers are competent to perform this function independently, others expressed uncertainty about their lack of familiarity with antiviral medications, implying a benefit to centralizing this function at a higher level. Similarly, several primary care providers disapproved of the protocol to have patients call a centralized telephone triage line for pandemic influenza-related questions, but concurrently lamented their lack of time in clinic for the increased number of pandemic-related patient visits, often from patients seeking information or reassurance. Given primary care providers’ constraints of time and/or clinical expertise, it is not unreasonable for countries to implement policies that consolidate certain pandemic-related clinical activities under the direction of a smaller number of trained clinical providers or public health officials. However, even when such policies are justified in order to facilitate consistency in implementation of clinical protocols, it is clear that some primary care providers will disagree with this disruption to clinical care, or to the perceived threat to their autonomy.

This article focuses on the pre-vaccination period, and thus did not address the role of primary care providers in administering vaccinations during the 2009 pandemic. It is unclear whether the views of primary care providers in these three countries views regarding vaccination responsibilities would have been more consistent. Regardless, vaccination represents a key topic of engagement and collaboration for primary care providers, public health officials, and emergency preparedness officials at the national, regional and local levels. Pandemic planning that incorporates a detailed scheme for identifying vaccination sites, delineating vaccine delivery protocols, and outlining emergency communication processes will stimulate conversation about what is feasible in the primary care setting, and how public health and emergency preparedness officials can adequately and efficiently inform and support their primary care partners. In turn, primary care providers will gain a clearer understanding of what to expect in a pandemic situation, and will have developed relationships with key partners, facilitating a mechanism for feedback as the arc of the pandemic unfolds.

Finally, this study calls into question the adequacy with which primary care is represented in public health/emergency preparedness planning and decision-making efforts, both prior to and during a pandemic situation. Some of the pandemic recommendations (e.g., required use of personal protective equipment, patient segregation) were perceived by providers as an ill fit with typical primary care practice, including the structure and function of the outpatient office setting. There is a critical need for decision-making entities to include representation of, or consultation with, practicing primary care providers who can speak to the feasibility of implementing proposed policies into day-to-day clinical practice. This need goes beyond having representatives of physician specialty organizations; rather, there should be a mechanism to solicit input from providers currently practicing in primary care (not hospital-based) settings. Documenting the composition of decision-making bodies, and exploring the link with primary care-appropriate policies, is an important area for future research.

## Conclusion

Primary care providers’ perspectives on, and satisfaction with, their pandemic roles and responsibilities are likely to reflect both the context of decision-making and communication during a pandemic situation, as well as the extent to which the designated primary care roles and responsibilities are feasible in the primary care setting. Involving practicing primary care providers in pandemic planning may help to articulate responsibilities that are feasible and acceptable to primary care providers and their public health partners. Central government purchase of personal protective equipment, vaccine supplies, and other materials necessary for primary care providers to carry out their assigned responsibilities is necessary to eliminate financial barriers to their participation in the pandemic response. Establishing and communicating the availability of dedicated information and communication channels for primary care providers will help to mitigate clinical uncertainty and inexperience and to ensure a more consistent response across primary care sites.

## References

[1] Kunin M, Engelhard D, Thomas S, Ashworth M, Piterman L (2015). Challenges of the Pandemic Response in Primary Care during the Pre-Vaccination Period. Israel Journal of Health Policy Research.

[2] Reynolds B, Deitch S, Schieber R. Crisis and emergency risk communication: Pandemic Influenza. Atlanta, GA: Centers for Disease Control and Prevention. Revised, October 2007. Available at: http://www.bt.cdc.gov/cerc/resources/pdf/cerc-pandemicflu-oct07.pdf

